# Small Molecule Immunomodulators as Next-Generation Therapeutics for Glioblastoma

**DOI:** 10.3390/cancers16020435

**Published:** 2024-01-19

**Authors:** Somaya A. Abdel-Rahman, Moustafa Gabr

**Affiliations:** 1 Department of Radiology, Molecular Imaging Innovations Institute (MI3), Weill Cornell Medicine, New York, NY 10065, USA; 2 Department of Medicinal Chemistry, Faculty of Pharmacy, Mansoura University, Mansoura 35516, Egypt

**Keywords:** small molecules, immune checkpoints, glioblastoma, drug discovery, immunomodulation, immunosuppression

## Abstract

**Simple Summary:**

Glioblastoma (GBM) is an aggressive brain tumor with limited treatment success. Despite efforts, the prognosis remains poor, impacting patients’ quality of life. Current therapies include surgery, radiation, and chemotherapy, but options for relapsed GBM are unclear. Immunotherapy shows potential, but the highly immunosuppressive nature of GBM poses challenges. Clinical trials with small-molecule inhibitors show mixed results, emphasizing the need for targeted immunomodulatory pathways in GBM treatment. This review explores potential strategies for improving GBM therapy through small molecule immunomodulators.

**Abstract:**

Glioblastoma (GBM), the most aggressive astrocytic glioma, remains a therapeutic challenge despite multimodal approaches. Immunotherapy holds promise, but its efficacy is hindered by the highly immunosuppressive GBM microenvironment. This review underscores the urgent need to comprehend the intricate interactions between glioma and immune cells, shaping the immunosuppressive tumor microenvironment (TME) in GBM. Immunotherapeutic advancements have shown limited success, prompting exploration of immunomodulatory approaches targeting tumor-associated macrophages (TAMs) and microglia, constituting a substantial portion of the GBM TME. Converting protumor M2-like TAMs to antitumor M1-like phenotypes emerges as a potential therapeutic strategy for GBM. The blood–brain barrier (BBB) poses an additional challenge to successful immunotherapy, restricting drug delivery to GBM TME. Research efforts to enhance BBB permeability have mainly focused on small molecules, which can traverse the BBB more effectively than biologics. Despite over 200 clinical trials for GBM, studies on small molecule immunomodulators within the GBM TME are scarce. Developing small molecules with optimal brain penetration and selectivity against immunomodulatory pathways presents a promising avenue for combination therapies in GBM. This comprehensive review discusses various immunomodulatory pathways in GBM progression with a focus on immune checkpoints and TAM-related targets. The exploration of such molecules, with the capacity to selectively target key immunomodulatory pathways and penetrate the BBB, holds the key to unlocking new combination therapy approaches for GBM.

## 1. Introduction

Glioblastoma (GBM), identified as the most aggressive diffuse glioma within the astrocytic lineage [1,2,3,4,5,6], is categorized as a grade IV glioma according to the World Health Organization (WHO) classification [7,8,9,10]. GBM constitutes approximately 57% of all gliomas and 48% of primary malignant central nervous system (CNS) tumors [1,2,3,4,5,6]. Although efforts towards multimodality GBM therapy have involved surgery, radiotherapy, and systemic treatments, the overall prognosis remains bleak [11,12,13,14,15,16]. Additionally, the associated morbidity, characterized by a progressive decline in neurologic function and quality of life, profoundly impacts patients, caregivers, and families. GBM treatment is intricate, initially involving a maximally safe surgical resection followed by radiation therapy and concurrent temozolomide chemotherapy [11,12,13,14,15,16]. Treatment options in the relapsed or recurrent stage of GBM lack a clear definition, with no established standard of care and limited evidence supporting interventions that extend overall survival [17,18]. Given that recent therapeutic advancements have only marginally increased the median survival to over 15 months in treated GBM patients [19,20,21,22,23], there is an urgent need to substantiate novel therapeutic strategies for GBM.

The immunotherapy revolution [24,25,26] holds potential to advance the development of more effective and well-tolerated treatments to combat the aggressive nature of GBM. Immunotherapy aims to leverage the body’s own immune system to recognize and eliminate abnormal tumor cells [27,28]. However, current trials with immunotherapies yield unsatisfactory outcomes in the majority of GBM patients [29,30,31,32,33,34,35,36], underscoring the need for a deeper understanding of the complex and dynamic interactions between glioma and immune cells. Regrettably, the benefits of immunotherapies for GBM have been confined to small randomized trials in the neoadjuvant setting and limited advantages in phase III trials in the adjuvant setting [29,30,31,32,33,34,35,36]. The highly immunosuppressive nature of GBM poses a significant challenge to immunotherapy, as glioma cells evade effective antitumor immunity by shaping the tumor microenvironment (TME) [37,38,39,40,41]. Consequently, there is an urgent need to develop new immunomodulatory approaches that can reverse immune suppression in GBM, unlocking the full potential of immunotherapy for this condition. In this context, the immunosuppressive tumor-associated macrophages (TAMs) and microglia comprise a substantial portion (30% to 50%) of the total tumor composition in GBM and play a crucial role in the GBM TME [42,43]. GBM TAMs originate from bone marrow (BM)-derived blood monocytes and brain-resident microglia [42,43]. Notably, monocyte-derived macrophages (MDMs) significantly contribute to the immunosuppressive microenvironment of high-grade glioma, indicating distinct functions of microglia and MDMs within the GBM TME [44,45]. Emerging evidence suggests that protumor M2-like TAMs are often accumulated and associated with higher-grade gliomas, while repolarizing TAMs toward an antitumor M1-like phenotype leads to tumor regression by producing proinflammatory cytokines and key molecules that stimulate T cell antitumor responses [46,47]. 

The impediment posed by the blood–brain barrier (BBB) in safeguarding brain tumor cells presents a challenge to the success of immunotherapy in GBM treatment [48,49]. The BBB, primarily consisting of CNS endothelial cells interconnected by intricate tight junctions with limited vesicular transport, hinders passive transport [50]. Only a select few low molecular weight or lipid-soluble molecules can traverse the BBB through passive transport [51]. Clinical evidence indicates that all GBM patients exhibit tumor regions with an intact BBB [52]. Consequently, effective drug delivery for GBM treatment must penetrate the BBB to target these regions or stimulate an endogenous cellular immune response capable of reaching them. Addressing these challenges has spurred extensive research endeavors focused on enhancing the BBB permeability of potential GBM therapies in preclinical models [53,54,55,56,57,58,59,60,61,62]. In contrast to monoclonal antibodies (mAbs) and other biologic-based therapeutics, small molecules can easily traverse the BBB and are more adaptable to pharmacokinetic optimization [63,64,65,66]. Consequently, identifying small molecules with favorable BBB permeability holds promise for GBM therapeutics with clinical potential. Up to now, over 200 clinical trials have been concluded or are currently underway for GBM [67]. In these trials, researchers employed small molecules either as standalone treatments or in combination regimens. The results of these trials indicated that small-molecule inhibitors, particularly kinase inhibitors, did not provide additional benefits for newly diagnosed GBM [68]. However, clinical investigations involving progressive GBM often asserted “noninferiority” in comparison to historical outcomes [67,68]. GBM drug candidates not only require high potency and selectivity against the intended therapeutic target, but also optimal brain penetration. Therefore, off-target and inadequate BBB penetration are major limitations of new therapeutics for GBM [69,70,71,72,73]. The development of small molecules that selectively target key immunomodulatory pathways in GBM and possess BBB permeability will enable the development of new combination therapy approaches for GBM. In this review, we discuss various immunomodulatory pathways implicated with GBM progression and highlight opportunities for therapeutic intervention using small molecules.

## 2. Immune Checkpoints

The immune system possesses a remarkable ability to balance T-cell activation and suppression, crucial for safeguarding the body against infections and cancer. However, an excessive T-cell response can result in chronic inflammation and tissue damage. To maintain this delicate equilibrium, the immune system induces the expression of co-inhibitory signals, known as immune checkpoints [74,75,76,77,78,79,80,81]. These surface molecules regulate host immunity when bound to their corresponding ligands or receptors. Cancer cells exploit this mechanism to evade T-cell destruction, leading to the development of immune checkpoint-targeting therapies aiming to restore T-cell anti-tumor immunity [74,75,76,77,78,79,80,81]. The success of immune checkpoint blockade in treating various malignancies has garnered significant attention in the past decade, earning recognition as the 2013 “Breakthrough of the Year” in Science magazine. Despite its success in various cancers, immune checkpoint blockade has shown limited therapeutic efficacy in GBM. Consequently, ongoing clinical trials focus on combination therapy, pairing checkpoint inhibitors with other therapeutic agents [82,83,84]. We highlight below key immune checkpoints that represent promising targets for GBM and have been subject to small molecule drug discovery research efforts.

### 2.1. PD-1/PD-L1

Programmed cell death protein 1 (PD-1) is a negative immune checkpoint that is mainly expressed on the surface of activated T cells and induces T-cell apoptosis upon binding to its ligand (PD-L1), expressed on the surface of tumor cells or antigen-presenting cells [85]. Clinical studies have revealed the expression of PD-L1 by tumor cells in GBM, and that PD-L1 expression level is correlated to the levels of malignancy and tumor aggressiveness [86,87]. However, monotherapeutic approaches based on PD-1/PD-L1 inhibition have resulted in poor outcomes in clinical trials, including GBM patients [88]. Moreover, anti-PD1 mAbs have been employed in combination therapy approaches for GBM. For example, the CheckMate-548 trial (NCT02667587) investigated the impact of incorporating nivolumab (anti-PD-1 mAb) into the established standard of care (temozolomide and radiation therapy) compared to a placebo combined with the standard care for a subset of GBM patients [89]. Regrettably, the CheckMate-548 trial did not achieve its primary objectives related to overall survival upon final analysis [89]. It is noteworthy to mention that anti-PD-1 therapies are approved for treating solid tumors characterized by microsatellite instability-high (MSI-H), mismatch repair deficiency (dMMR), or high tumor mutation burden (TMB-H), including GBM [90]. This highlights the potential of PD-1-targeted agents as potential GBM therapies.

**Figure 1:**
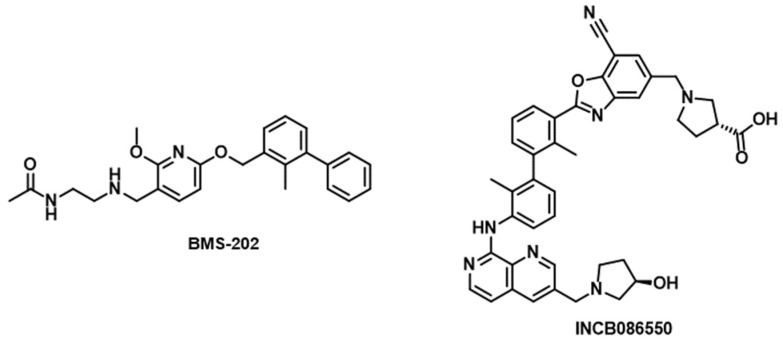
Chemical structures of BMS-202 and INCB086550.

There are limited instances of successful discovery of small molecule PD-1/PD-L1 inhibitors. Bristol Myers Squibb (BMS) researchers have unveiled a series of substituted biphenyl derivatives, showcasing their effectiveness in impeding PD-1/PD-L1 signaling [91]. A representative example of the BMS compounds is BMS-202 (Figure 1). Various companies, including Incyte Corporation, have identified a variety of small molecule PD-1/PD-L1 inhibitors utilizing the biphenyl core [91,92]. The most notable success in this regard is the development of INCB086550 (Figure 1) with potent immunomodulatory activity in animal immuno-oncology models that warranted advancement to an ongoing phase I clinical trial [93]. Remarkably, numerous research groups have endeavored to optimize the BMS compounds as PD-L1/PD-1 inhibitors [94,95,96,97,98]. 

### 2.2. TIM-3

**Figure 2:**
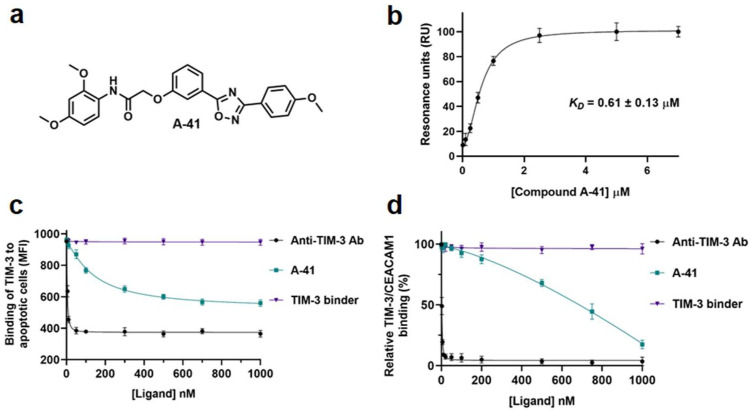
(**a**) Chemical structure of **A-41**; (**b**) SPR binding curve of compound **A-41** to TIM-3 [102]—error bars represent standard deviation (n = 3); (**c**) Detection of the binding of TIM-3 to apoptotic Jurkat T cells expressing surface PtdSer using flow cytometry (MFI) upon preincubation with different concentrations of the tested agents (anti-TIM-3 Ab (M6903), **A41**, and small molecule TIM-3 binder) [102]; (**d**) Binding of His-tagged CEACAM1 to plate-bound TIM-3 in competitive ELISA upon preincubation with different concentrations of the tested agents (anti-TIM-3 Ab (M6903), **A41**, and a small molecule TIM-3 binder) [102]. Error bars represent standard deviation (n = 5).

T-cell immunoglobulin and mucin-domain containing-3 (TIM-3) is a key negative immune checkpoint that is expressed on various immune cells, including T cells, B cells, natural killer cells, and dendritic cells [99]. The immunomodulatory function of TIM-3 is based on key interactions with TIM-3 ligands (e.g., galectin-9, phosphatidylserine (PtdSer), and carcinoembryonic antigen-related cell adhesion molecule 1 (CEACAM1)) expressed on tumor cells or antigen-presenting cells [99]. The higher intertumoral expression of TIM-3 in GBM in comparison to low-grade gliomas in CD4^+^ and CD8^+^ T cells is indicative of the contribution of TIM-3 to glioma severity in patients [100]. Moreover, anti-TIM-3-targeted therapeutics have revealed promising outcomes in combination therapy approaches in preclinical models of GBM [101]. Our lab has recently utilized a virtual screening approach to identify a first-in-class small molecule TIM-3 inhibitor [102]. The top optimized lead compound from our study (**A-41**, Figure 2a) binds TIM-3 with submicromolar binding affinity, based on surface plasmon resonance (SPR) screening (Figure 2b), and inhibits key TIM-3 interactions with PtdSer and CEACAM1 (Figure 2c,d) [102]. Further optimization of our TIM-3 lead (**A-41**) will result in promising compounds that can be incorporated into combination therapy approaches in preclinical models of GBM.

### 2.3. LAG-3

**Figure 3:**
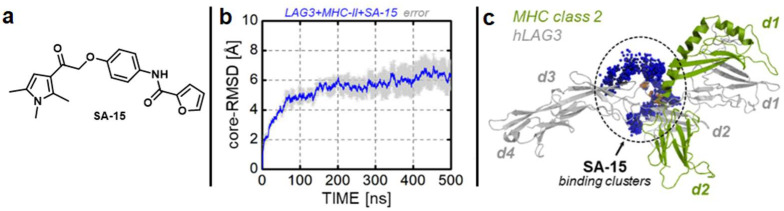
(**a**) Chemical structure of **SA-15**; (**b**) Core-RMSD graph of the dynamics of LAG-3/MHCII in complex with compound **SA-15**, the blue color indicates the average core-RMSD of the system, while the gray transparency represents the error [105]. (**c**) The cluster for compound **SA-15** binding to the LAG-3/MHCII interface [105].

Lymphocyte-activation gene 3 (LAG-3) is a negative immune checkpoint that is expressed on various immune cells and inhibits T cell activation via binding major histocompatibility complex class II (MHC-II) [103]. The immunosuppression function of LAG-3 is achieved in conjunction with other immune checkpoints, such as PD-1 [103]. By employing LAG-3 knockout mice, Lim and colleagues validated LAG-3 inhibition as an efficient strategy to control GBM growth [104]. LAG-3 was established as an early marker of exhaustion of effector T cells, providing the basis for the potential of early LAG-3 inhibition to release an antitumor immune response [104]. Remarkably, a combination of LAG-3 and PD-1 biologics has the ability to eradicate GBM in vivo [104]. It is noteworthy to mention that anti-LAG-3 mAb (BMS-986016) has advanced to a phase I clinical trial for GBM in combination with PD-1 blockade (NCT02658981). In 2023, our group reported the first examples of small molecule LAG-3 inhibitors that can be pursued as drug candidates for GBM in preclinical studies [105,106]. The most promising compound from our screening campaign (**SA-15**, Figure 3a) inhibits key LAG-3 interactions in cell-free and cell-based assays [105]. We further investigated the binding mode of **SA-15** to LAG-3 using molecular dynamics (MD) simulation (Figure 3b,c [105]), providing the mechanistic assessment of the LAG-3/MHCII inhibition by **SA-15** [105]. These computational studies will facilitate hit-to-lead optimization efforts in the future to develop potent leads with nanomolar LAG-3 inhibition profiles. We anticipate that such discoveries will pave the way for incorporating small molecule LAG-3 inhibitors in combination therapy regimens in preclinical and clinical evaluations for GBM treatment.

### 2.4. CTLA-4

**Figure 4:**
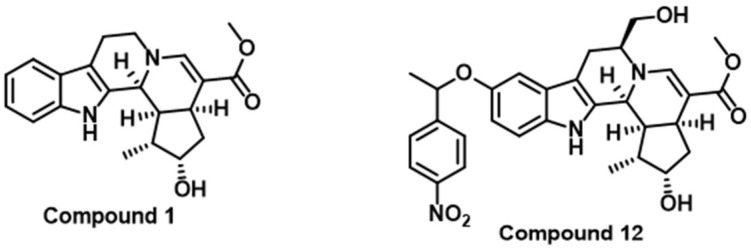
Chemical structures of pentacyclic indole alkaloid-like compounds as small molecule CTLA-4 inhibitors [110].

The cytotoxic T-lymphocyte-associated antigen 4 (CTLA-4, CD152) is a co-inhibitory receptor that binds to cell surface ligands CD80 and CD86 on the antigen-presenting cells [107]. Given its ability to disrupt CD28 co-stimulatory signaling, CTLA-4 can inhibit T-cell antigen-specific responses, establishing itself as a key negative regulator of T-cell activation [107]. Elevated CTLA-4 expression in high-grade gliomas compared to low-grade gliomas suggests a positive correlation between CTLA-4 expression and cancer severity [108]. A phase II clinical study is presently investigating the efficacy of anti-CTLA-4 in GBM patients post-radiation and chemotherapy, comparing temozolomide treatment alone with temozolomide combined with ipilimumab (anti-CTLA-4) [109]. Several concurrent clinical trials are exploring the combined efficacy of CTLA-4 with PD-1 in GBM treatment, aiming to maximize the potential of anti-CTLA-4 therapy. Efforts to develop CTLA-4-targeted small molecules have included the discovery and optimization of pentacyclic indole alkaloid-like compounds (Figure 4) as dual CTLA-4 and PD-1 small molecule inhibitors with the ability to suppress the CTLA-4 and PD-L1 gene expression and their protein expression on the cell surface [110].

### 2.5. TIGIT

The T-cell immunoreceptor with Ig and ITIM domain (TIGIT) is a co-inhibitory receptor expressed in lymphocytes [111]. TIGIT has recently emerged as a promising target for immunotherapy of GBM. Preclinical work validated the ability of anti-TIGIT mAbs to effectively inhibit T-cell proliferation and enhance the anti-tumor immune response, either as a standalone therapy or in conjunction with other immune checkpoint inhibitors [112]. In GBM patients, the significant upregulation of TIGIT expression in CD8^+^ T cells at the tumor site compared to healthy individuals has been reported [113]. In agreement with this finding, a correlation between increased expression of the TIGIT ligand poliovirus receptor (PVR) and reduced survival in glioma patients was established [114]. Remarkably, a combination of anti-TIGIT with anti-PD-1 mAbs led to improved survival rates compared to monotherapy in a murine GBM model [114]. This improvement was associated with elevated effector T-cell activity and the downregulation of regulatory T cells [114]. Various virtual screening campaigns have resulted in the identification of TIGIT-targeted small molecules with potent in vitro and in vivo immunomodulatory activity [115,116]. These recent discoveries underscore the potential of TIGIT-targeted small molecules for future development as potential GBM therapies.

### 2.6. VISTA

**Figure 5:**
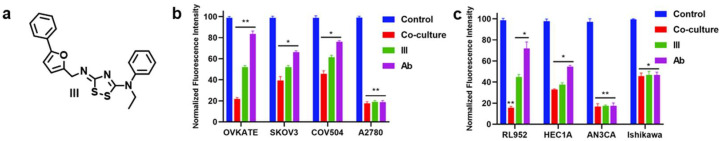
(**a**) Chemical structure of **III**; PrestoBlue viability of Jurkat T-cells in the presence of III (5 μM) and VISTA Ab (1 μM) upon co-culturing with ovarian (**b**) and endometrial (**c**) cancer cell lines. Error bars represent standard deviation (n = 3). (* *p* < 0.05; ** *p* < 0.01 relative to untreated control) [119].

V-domain immunoglobulin suppressor of T cell activation (VISTA) is a negative immune checkpoint that shares sequence homology with PD-L1 and has a dual ability to function as a receptor on T cells or a ligand on antigen-presenting cells [117]. Clinical data reveal the correlation between high levels of VISTA expression and poor prognosis in glioma patients, especially in grade III/IV gliomas [118]. Thus, increasing research interest has been directed toward VISTA as a potential target for advanced gliomas. We utilized random screening of a chemical library using a fluorescence-based assay to identify small molecule VISTA inhibitors [119]. Subsequent hit-to-lead optimization guided by molecular docking, saturation transfer difference (STD) NMR, and site-directed mutagenesis led to the discovery of a small molecule VISTA inhibitor (**III**, Figure 5a) with submicromolar VISTA binding affinity [119]. Importantly, **III** demonstrated the ability to restore T-cell proliferation in the presence of VISTA-expressing ovarian and endometrial cancer cell lines (Figure 5b,c) [119]. Ongoing efforts in our lab are focused on the optimization of **III** to afford leads with optimal properties for BBB permeation that can be evaluated in preclinical models of GBM.

### 2.7. ICOS

**Figure 6:**
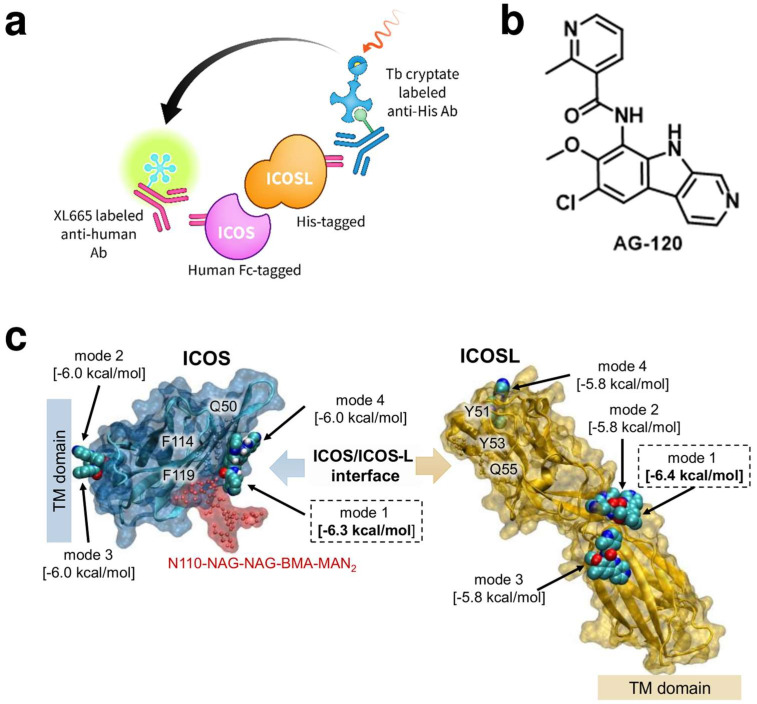
(**a**) Schematic illustration of our developed fluorescence-based assay to identify ICOS/ICOSL inhibitors [122]; (**b**) Chemical structure of **AG-120**; (**c**) The highest-scored position of **AG-120** in ICOS and ICOSL obtained in docking studies. Values provided in brackets correspond to binding affinity determined based on the scoring function [122].

The inducible co-stimulator (ICOS) belongs to the costimulatory molecule family, which includes ICOS, CD28, and CTLA-4 [120]. It is highly expressed on the surface of activated and mature T cells rather than on naïve T cells [120]. The activation of the costimulatory signal occurs when ICOS engages with its specific ligand (ICOSL), leading to the facilitation of various immune-related processes. These processes include the development of germinal centers, activation of T-cell-dependent B cells, and the switch of antibody class [120]. Significantly, the ICOS/ICOSL pathway plays a crucial role in promoting the differentiation, proliferation, activation, and survival of T cells themselves [120]. Moreover, ICOS contributes to the enhanced secretion of various immune cytokines. Any deviation in ICOS expression can result in a spectrum of pathophysiological dysfunctions, including immunodeficiency, susceptibility to opportunistic infections, and the development of malignant tumors [120]. Increased ICOS levels in patients demonstrate a correlation with greater glioma malignancy and a significant association with regulatory T cell (Treg) activity within the immune responses related to gliomas [121]. In the analysis of cell lineage, gliomas with elevated ICOS levels exhibited a tendency to recruit dendritic cells, monocytes, and macrophages into the TME [121]. Thus, the development of ICOS-targeted drug candidates has the potential to pave the way for new immunomodulatory therapeutic strategies for GBM. We recently developed and optimized a fluorescence-based assay (Figure 6a) for high-throughput screening (HTS) of chemical libraries to identify small molecule ICOS/ICOSL inhibitors [122]. Implementation of our HTS assay in screening a focused chemical library resulted in the identification of **AG-120** (Figure 6b) as a first-in-class small molecule ICOS/ICOSL inhibitor [122]. Our computational study validated the ability of **AG-120** to interact with ICOS, sterically gate ICOSL binding, and prevent ICOS/ICOSL complex formation (Figure 6c) [122].

## 3. TAM-Related Targets

### 3.1. CCL2/CCR2 Axis

CCR2 is a member of the human Class A G protein-coupled receptors (GPCRs) within the chemokine receptor subfamily [123]. Two isoforms, namely CCR2A and CCR2B, exhibit variations in their C-terminal regions, leading to distinct signaling properties. Notably, CCR2 finds expression in monocytes/macrophages in humans [123]. CCL2 serves as the prototypical chemokine that binds to CCR2, and the interaction between CCL2 and CCR2 is the most significant for the functionality of CCR2 [123]. Activation of the chemokine receptor CCR2 through ligation initiates the activation of various downstream signaling pathways [123]. Inhibition of the TAM infiltration into the GBM TME by targeting the interactions between chemo-attractants and their receptors is a promising therapeutic strategy for GBM. Thus, CCL2/CCR2 represents a potential target to overcome the immunosuppressive nature of the GBM TME. CCL2, secreted by cancer cells, attracts myeloid cells expressing CCR2 (such as TAMs and myeloid-derived suppressor cells) to the TME of GBM [124]. In preclinical models of GBM, CCR2 blockade via an antagonist suppresses the recruitment of TAMs and improves the efficacy of immune checkpoint inhibitors [124]. Although numerous small molecule CCR2 antagonists have been developed in recent years [125], the lack of clinical efficacy requires further investigation into their mechanism of action. Assessment of the binding sites of these small molecule antagonists on CCR2 has revealed that multiple binding sites are present on CCR2, which is a key determinant of the different modes of inhibition of these compounds [125]. Nevertheless, small molecule-based CCR2 blockade has the potential to maximize the number of GBM patients benefiting from immunotherapies. This potential is exemplified by the ability of a small molecule inhibitor for CCR2 (CCR2i) to maximize the efficacy of PD-1 blockade in a murine model of a cutaneous T-cell lymphoma (CTCL) [126]. 

### 3.2. CHI3L1/Gal-3

**Figure 7:**
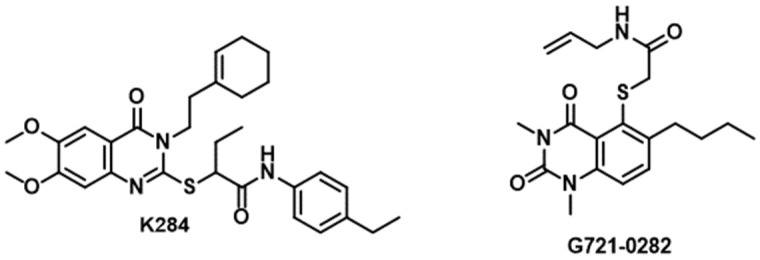
Chemical structures of **K284** and **G721-0282**.

Chitinase-3 like-protein-1 (CHI3L1) is a secreted glycoprotein categorized under the glycoside hydrolase family 18 that plays a critical role in shaping the landscape of the GBM TME. Notably, CHI3L1 has received growing research interest as a novel prognostic marker for high-grade gliomas [127]. Clinically, increased mRNA level of CHI3L1 has been associated with poor survival of GBM patients [128]. Moreover, CHI3L1 has been identified as a regulator of glioma cell invasion, migration, and growth [129]. Additionally, it plays a role in driving tumor vascularization in glioblastoma-stem-like cells (GSCs) [130]. Furthermore, CHI3L1 promotes tumorigenesis in GSCs with unmethylated MGMT promoter and contributes to temozolomide resistance [131]. A key aspect of the ability of CHI3L1 to modulate the GBM TME is based on its ability to form a protein complex with galectin-3 (Gal3) or galectin-3–binding protein (Gal3BP) to promote macrophage-mediated immune suppression [132]. The interaction between CHI3L1 and Gal-3 promotes the infiltration of monocyte-derived macrophages (MDMs) and induces their reprogramming into a tumor-promoting M2-like phenotype [132]. Notably, this process is negatively regulated by Gal3BP. The CHI3L1/Gal-3 pathway facilitates GBM evasion from immune surveillance by leveraging the CHI3L1/Gal-3 protein complex capacity to activate the AKT/mTOR-mediated transcriptional regulatory network (involving NF-κB and CEBPβ) [132]. This activation results in a macrophage switch from immune stimulation to immune suppression [132]. These findings provide a basis for the disruption of the CHI3L1/Gal-3 protein complex as a potential strategy to mitigate tumor immunosuppression and enhance the antitumor immune response within the GBM tumor microenvironment. In a proof-of-concept study published in 2021, the local delivery of a peptide-based inhibitor targeting CHI3L1/Gal-3 (Gal3BP mimetic peptide) into brain tumors demonstrated the ability to induce tumor regression in treated mice. This effect was accompanied by a decrease in M2-like macrophages and an increase in M1-like macrophages and CD8^+^ T cells within the TME [132]. The recent discovery of small molecule CHI3L1 inhibitors, such as **K284** and **G721-0282** (Figure 7), will remarkably contribute to realizing the potential of CHI3L1 as a therapeutic target for GBM [133,134].

### 3.3. SLIT2/ROBO

The Slit guidance ligands (SLITs) constitute a group of secreted proteins that orchestrate positional interactions between cells and their environment during development. They achieve this function by signaling through the roundabout (ROBO) receptors [135]. In mammals, SLITs 1, 2, and 3 transmit signals by binding to the second leucine-rich repeat region (D2), specifically interacting with the Ig1 domain of ROBO1 and ROBO2 receptors [136]. The SLIT2/ROBO pathway assumes pivotal roles in organ development, homeostatic maintenance, and the process of tumorigenesis [135]. In the context of tumors, SLIT2 possesses a proangiogenic role, and elevates tumor cell aggressiveness and migration, metastatic spread, and resistance to therapeutics [137,138]. Recently, the SLIT2/ROBO signaling pathway was identified as a novel immune evasion mechanism within the TME of GBM [139]. Elevated SLIT2 expression observed in GBM patients and mouse models led to the accumulation of immunosuppressive TAMs and vascular abnormalities [139]. Notably, when SLIT2 was knocked down in glioma cells, or its systemic inhibition was achieved through an SLIT2-trapping protein (ROBO1Fc), it prevented the tumor-promoting polarization of TAMs and the expression of angiogenic genes [139]. This intervention resulted in improved functionality of tumor vessels and enhanced efficacy of chemotherapy and immunotherapy in GBM mouse models [139]. Remarkably, the impact of SLIT2 inhibition on angiogenesis and the augmentation of T cell response surpasses the outcomes achieved by previously explored therapeutic approaches targeting the TAM component within the GBM TME [139]. As of now, there are no ongoing clinical trials evaluating the inhibition of SLIT2/ROBO as a therapeutic strategy for GBM. Therefore, there is an urgent need to direct research efforts towards small molecule-based SLIT2/ROBO inhibition.

### 3.4. CD47

**Figure 8:**
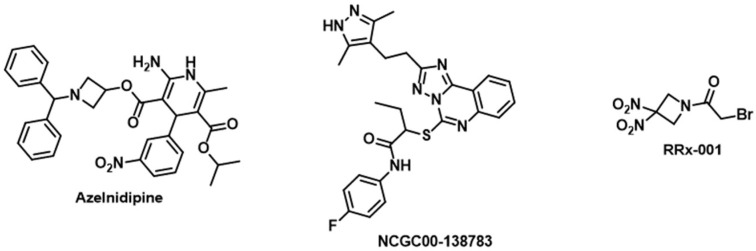
Chemical structures of small molecule CD47 inhibitors.

The cluster of differentiation (CD) 47, present on both healthy and malignant cells, regulates macrophage-mediated phagocytosis by transmitting a “don’t eat me” signal to the signal regulatory protein alpha (SIRPα) receptor [140]. Growing evidence supports the notion that blocking the interaction between CD47 and SIRPα can augment the clearance of cancer cells by macrophages [140]. Furthermore, inhibiting the CD47/SIRPα interaction has the potential to enhance antigen cross-presentation, promoting T-cell priming and activating an adaptive antitumor immune response. Consequently, targeting the CD47/SIRPα axis holds significant promise for advancing tumor immunotherapy. Research on CD47 mAbs is currently at the forefront, yielding impressive results. However, a notable challenge has emerged in the form of hematotoxicity, particularly anemia, which has become the most prevalent adverse effect associated with CD47 monoclonal antibody treatment [140]. To address this issue, more specific targeted drugs, including bispecific antibodies, SIRPα/Fc fusion protein antibodies, and small-molecule inhibitors, have been developed with the aim of mitigating hematotoxicity while maintaining therapeutic efficacy [140]. Depleting CD47 in GBM cells has shown a significant increase in macrophage phagocytosis and inhibition of GBM tumor growth [141]. This underscores the therapeutic potential of targeting the CD47/SIRPα axis in GBM patients. Preclinical studies utilizing humanized anti-CD47 antibodies have demonstrated promising antitumor effects in pediatric glioma patient-derived xenograft models [142]. Significant research efforts have been directed towards the discovery and optimization of small molecule CD47 inhibitors over the past years. For example, a virtual screening campaign resulted in the identification of Azelnidipine (Figure 8) as a CD47/SIRPα inhibitor and potent in vivo inhibitory activity against the growth of CT26 tumors [116]. **NCGC00-138783** (Figure 8) is another example of a small molecule that directly binds CD47 and blocks CD47/SIRPα interaction [143]. The discovery of **NCGC00-138783** was made possible based on the implementation of a homogenous CD47/SIRPα cell-based, ligand-based assay [143]. Moreover, **RRx-001** (Figure 8) is an anticancer drug candidate in phase III clinical trials, which inhibits CD47 expression and polarizes tumor-associated macrophages from a low phagocytic M2 phenotype to a high phagocytic M1 phenotype [144].

### 3.5. CSF-1/CSF-1R

The colony-stimulating factor-1 receptor (CSF-1R), also identified as the macrophage colony-stimulating factor (M-CSF) receptor, is a transmembrane tyrosine kinase receptor located on the cell surface of various cell types, including microglial cells, bone-marrow-derived macrophages, monocytes, osteoclasts, and dendritic cells [145]. The CSF-1/CSF-1R axis plays a crucial role in governing the survival, proliferation, differentiation, and functions of mononuclear phagocytes [145]. In human high-grade gliomas, there is an overexpression of the cytokine CSF1 and its corresponding receptor CSF1R in microglia and macrophages [146]. Using the immunocompetent GBM preclinical mode, CSF-1R inhibition improved the response to radiotherapy, validating its potential as a therapeutic strategy to maximize radiotherapy-induced antitumor immune responses [147]. In past years, there has been a notable surge in efforts focused on the discovery of small molecule CSF-1R inhibitors, fueled in part by the approval of pexidartinib in 2019 for treating tenosynovial giant cell tumors [148]. This increased activity is evident in both the publication of journal articles and the filing of patent applications centered around small molecule inhibitors of CSF-1R [148]. A remarkable aspect of this research is the unexpected diversity of chemical classes that have demonstrated potency and selectivity as CSF1-R inhibitors [147]. Furthermore, these inhibitors have shown promise in addressing a wide range of disease states, including cancer, arthritis, and conditions associated with ‘cytokine storm’ syndromes [148]. 

### 3.6. IL-6/IL-6R

Interleukin-6 (IL-6) is a cytokine overexpressed during inflammation as an acute-phase response and signals through binding its unique receptor alpha subunit, IL-6Rα, located on the plasma membrane [149]. It has been documented that IL-6 signaling plays a critical role in a variety of cancers, as demonstrated by the elevated expression levels of IL-6 mRNA and protein in colorectal, prostate, breast, ovarian, pancreatic, and cervical cancer [149]. Multiple studies indicate a correlation between the expression of IL-6 and glioma tumor grade, as well as overall patient survival [150]. Specifically, the mRNA expression of IL-6 was notably higher in GBM patient samples compared to samples from individuals with lower histopathological grades [150]. IL-6 signaling plays a pivotal role in promoting various activities that support gliomagenesis, including cell invasion and migration. This contributes to the invasive nature of glioblastoma, leading to reduced treatment efficacy and high rates of recurrence. Studies have demonstrated that STAT3 activation, induced by IL-6 signaling, specifically promotes cell invasion and migration in U251 and T98G glioblastoma cells [151]. Remarkably, an antibody cocktail-based immunotherapy that combines checkpoint blockade with dual-targeting of IL-6 and CD40 resulted in the extension of animal survival in two syngeneic GBM mice models [152]. The extensive focus on the discovery and preclinical evaluation of small molecule IL-6 inhibitors in recent years [153,154,155] holds promise for the future of small molecule-based IL-6/IL-6R inhibition in GBM treatment.

## 4. Conclusions

The current clinical landscape of small molecule immunomodulators underscores their pivotal role in reshaping therapeutic approaches. In Table 1, we provide a summary of key small molecule immunomodulators discussed in this article. These compounds, designed to intricately influence immune responses, are currently at various stages of preclinical and clinical testing. However, the journey from laboratory success to clinical application poses significant challenges. Fine-tuning these small molecules to ensure precise immune modulation without adverse effects faces various hurdles. Issues related to bioavailability, pharmacokinetics, and potential off-target effects necessitate meticulous consideration. Overcoming these challenges is crucial for unlocking the full therapeutic potential of small molecule immunomodulators, paving the way for innovative and targeted interventions in the realm of immunotherapy. 

GBM poses persistent challenges to effective treatment despite extensive efforts in multimodal therapies. While immunotherapy has emerged as a promising avenue, its success is hampered by the intricate and highly immunosuppressive microenvironment within GBM. A pivotal focus on immunomodulation, particularly targeting immune checkpoints and TAMs, offers potential by transitioning the microenvironment from immunosuppressive to anti-tumor. However, the BBB presents a key obstacle, necessitating the development of small molecules capable of selectively targeting GBM while crossing the BBB. Current clinical trials, notably with kinase inhibitors, are yielding varied results, underscoring the need for further research in this domain. Overcoming off-target effects and enhancing BBB penetration are critical for the successful development of small molecule immunomodulators as potential therapies for GBM. Navigating the intricate landscape of GBM treatment requires a comprehensive understanding of the immunosuppressive microenvironment and advancements in small molecule therapeutics. This review discussed the ongoing challenges and opportunities, emphasizing the importance of continued research to uncover novel strategies for combating GBM. We highlighted key checkpoints and TAM-related targets that were subject to small molecule drug discovery research. The discussion involved our recently reported first-in-class small molecule inhibitors of various checkpoints. These examples represent promising starting points to develop small molecule immunomodulators for GBM.

## Figures and Tables

**Table 1:** Summary of small molecule immunomodulators discussed in this review.

Compound	Target	Mechanism
**BMS202**	PD-L1	Directly binds PD-L1 and induces PD-L1 dimerization [91]
**INCB086550**	PD-L1	Induces PD-L1 dimerization and internalization, resulting in blocking PD-L1/PD-1 [93]
**A-41**	TIM-3	Directly binds TIM-3 and blocks TIM-3/ligand interaction [102]
**SA-15**	LAG-3	Directly binds LAG-3 and blocks key LAG-3 interactions [105]
**Compound 1**	CTLA-4	Inhibits CTLA-4 gene expression [110]
**Compound 12**	CTLA-4 and PD-L1	Suppresses CTLA-4 and PD-L1 gene expression as well as protein expression on cell surface [110]
**III**	VISTA	Binds VISTA and blocks key VISTA interactions [119]
**AG-120**	ICOS	Binds near the ICOS/ICOSL interface and inhibits the interaction [120]
**K284**	CHI3L1	Binds CHI3L1 and prevents the binding of CHI3L1 to its receptor [133]
**G721-0282**	CHI3L1	Decreases the chronic unpredictable mild stress-elevated levels of CHI3L1 [134]
**Azelnidipine**	CD47 and TIGIT	Dual inhibitor of CD47/SIRPα and TIGIT/PVR interactions [116]
**NCGC00-138783**	CD47	Blocks CD47/SIRPα interaction in cell-based assay [143]
**RRx-001**	CD47	Decreases the expression levels of CD47 and SIRPα on tumor cells and monocytes/macrophages, respectively [144]

## Data Availability

Data can be found within the article.
